# Leakage Mechanism and Cycling Behavior of Ferroelectric Al_0.7_Sc_0.3_N

**DOI:** 10.3390/ma17020397

**Published:** 2024-01-12

**Authors:** Li Chen, Qiang Wang, Chen Liu, Minghua Li, Wendong Song, Weijie Wang, Desmond K. Loke, Yao Zhu

**Affiliations:** 1 Institute of Microelectronics, Agency for Science, Technology and Research (A*STAR), Singapore 138634, Singapore; chen_li@ime.a-star.edu.sg (L.C.); li_minghua@ime.a-star.edu.sg (M.L.); wang_weijie@ime.a-star.edu.sg (W.W.); 2 Department of Science, Mathematics and Technology, Singapore University of Technology and Design, Singapore 487372, Singapore; wang_qiang@ihpc.a-star.edu.sg

**Keywords:** AlScN, ferroelectric, leakage, cycling, P-F emission, DFT calculation

## Abstract

Ferroelectric scandium-doped aluminum nitride (Al_1-x_Sc_x_N) is of considerable research interest because of its superior ferroelectricity. Studies indicate that Al_1-x_Sc_x_N may suffer from a high leakage current, which can hinder further thickness scaling and long-term reliability. In this work, we systematically investigate the origin of the leakage current in Al_0.7_Sc_0.3_N films via experiments and theoretical calculations. The results reveal that the leakage may originate from the nitrogen vacancies with positively charged states and fits well with the trap-assisted Poole-Frenkel (P-F) emission. Moreover, we examine the cycling behavior of ferroelectric Al_0.7_Sc_0.3_N-based FeRAM devices. We observe that the leakage current substantially increases when the device undergoes bipolar cycling with a pulse amplitude larger than the coercive electric field. Our analysis shows that the increased leakage current in bipolar cycling is caused by the monotonously reduced trap energy level by monitoring the direct current (DC) leakage under different temperatures and the P-F emission fitting.

## 1. Introduction

The substantial increase in data generation and processing associated with the recent rise of artificial intelligence (AI), machining learning (ML), and Internet-of-Things (IoT) has created new challenges in improving computing efficiency and speed. Using an in-memory computing architecture to reduce or eliminate the data movement between the memory and processor unit can save significant power while increasing throughput [1,2,3,4,5]. Extensive studies to construct the building blocks for in-memory computing have been conducted, primarily consisting of non-volatile memory (NVM) devices, such as resistive random-access memory (RRAM) [6,7,8,9,10], ferroelectric random-access memory (FeRAM) [11], ferroelectric field-effect transistor (FeFET) [12,13,14], phase-change memory (PCM) [15], and magnetoresistive random-access memory (MRAM) [16]. Among these, ferroelectric memory devices relying on the switching polarization states of ferroelectric materials possess the advantages of ultra-fast speed and ultra-low power consumption, offering great potential for in-memory computing.

The wurtzite-structure, scandium-doped aluminum nitride (Al_1-x_Sc_x_N) is a widely studied piezoelectric material that has improved piezoelectricity compared with undoped aluminum nitride (AlN) [17,18]. The recent discovery of ferroelectricity in Al_1-x_Sc_x_N offers promise in ferroelectric memory applications [19,20,21,22,23,24,25,26,27]. Unlike other conventional ferroelectric materials, Al_1-x_Sc_x_N exhibits large remnant polarization (*P*_r_) of >100 µC/cm^2^, sub-10 nm scaling capability, and an ideal square-shape, polarization-electric field (*P*-*E*) hysteresis. Additionally, the coercive field (*E*_c_) of Al_1-x_Sc_x_N strongly depends on the Sc doping concentration, in which a higher Sc doping concentration leads to a lower *E*_c_ and can help reduce the operating voltage of Al_1-x_Sc_x_N-based memory devices [19]. However, the leakage current of Al_1-x_Sc_x_N with an even higher Sc doping concentration may become larger. Therefore, a moderate Sc doping concentration of ~30% is desired for achieving a moderate *E*_c_ and leakage current that enables the implementation in memory applications. Furthermore, it is CMOS-BEOL (back-end-of-line) compatible with a deposition temperature below 400 °C, and the process technology has been well established for MEMS resonator, filter, and transducer applications [28,29,30]. To date, Al_1-x_Sc_x_N-based ferroelectric memory devices, such as a ferroelectric field-effect transistor and a metal-ferroelectric-metal (MFM) diode, have been successfully demonstrated [31,32]. However, Al_1-x_Sc_x_N suffers from an undesired leakage current and poor endurance, which are major obstacles to practical implementation. Although the leakage and cycling behavior of Al_1-x_Sc_x_N has been characterized, the origin of the high leakage is still under debate [33,34,35,36,37]. In our previous works, the conduction mechanism, origin of leakage current, and cycling behavior have been preliminarily studied. The results showed that the dominant conduction mechanism is trap-assisted Poole–Frenkel (PF) emission and the type of vacancies leading to large leakage is a nitrogen vacancy instead of aluminum or scandium vacancies [36], and the bipolar cycling would cause grain fragmentation [37]. However, the trap energy level (*Φ*_T_), specific charge state of the vacancy, and impact of cycling on leakage current have not been deeply investigated yet. Therefore, systematic studies of the underlying mechanisms for the leakage and cycling behavior are still needed for further ultra-thin film growth and device design optimization.

In this paper, we deeply investigate the reliability of the Al_0.7_Sc_0.3_N by studying the origin of leakage and cycling behavior of ferroelectric Al_0.7_Sc_0.3_N-based FeRAM devices by both experimental and theoretical methods. Based on the experimental results, the as-grown Al_0.7_Sc_0.3_N is identified as a p-type material by P-F emission fitting with the consideration of compensation factor, which is an essential factor to identify the type of material and extract the *Φ*_T_. Moreover, it is found that the large leakage of the p-type Al_0.7_Sc_0.3_N can be attributed to nitrogen vacancies with positively charged states via theoretical calculation. Furthermore, the cycling tests show that leakage substantially increases after the FeRAM devices undergo bipolar cycling pulses with the amplitude larger than the *E*_c_, while the leakage current shows no obvious increment after either unipolar cycling pulses or bipolar cycling pulses with the amplitude smaller than the *E*_c_. This observation indicates that the leakage increment requires the polarization to be switched. At the same time, the evolution of the DC leakage under different temperatures is monitored and analyzed via P-F emission fitting, showing that the *Φ*_T_ decreases from 0.69 eV to 0.44 eV after 10^5^ bipolar cycling and eventually leads to a further leakage increment.

## 2. Experiment and Methods

**Figure 1:**
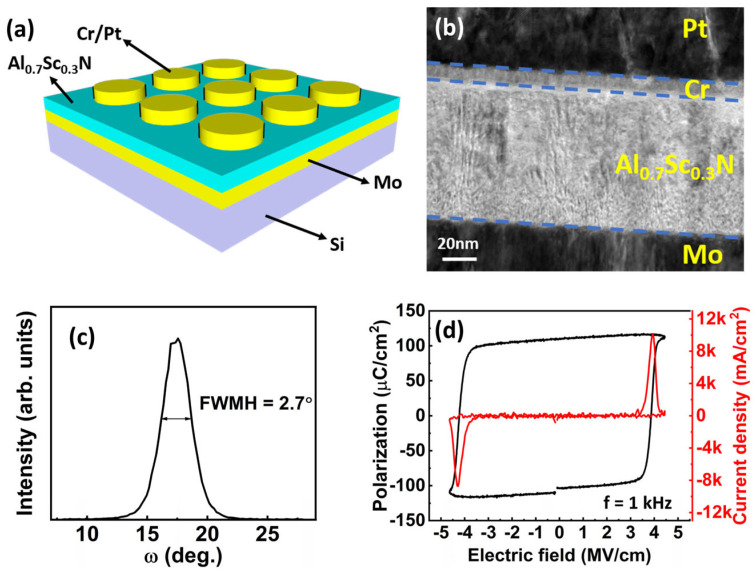
(**a**) Schematic of the Al_0.7_Sc_0.3_N-based MFM capacitor array. (**b**) TEM image of the MFM stack. (**c**) X-ray diffraction rocking curve of the (002) reflection of the Al_0.7_Sc_0.3_N film. (**d**) Representative *P-E* loop and dynamic current loop of the Al_0.7_Sc_0.3_N-based MFM capacitor.

The Al_0.7_Sc_0.3_N-based MFM capacitors were prepared on an 8-inch silicon wafer. A 100 nm thick Al_0.7_Sc_0.3_N ferroelectric film was deposited by pulsed laser deposition (PLD), and patterned on the 200 nm molybdenum (Mo) layer, followed by defining 10 nm chromium (Cr) and 100 nm platinum (Pt) layers as the top circular electrodes with radii ranging from 20 to 100 µm (Figure 1a). The transmission electron microscope (TEM) photo of the cross-section is plotted in Figure 1b. The peak in the X-ray diffraction (XRD) rocking curve for the (002) reflection of the Al_0.7_Sc_0.3_N film is observed with the full-width half-maximum (FWHM) value of 2.7° (Figure 1c), indicating a good *c*-axis orientation. The as-fabricated capacitor exhibits clear ferroelectricity with a large *P*_r_ > 100 µC/cm^2^ and a steep switching slope (Figure 1d).

The ferroelectric behavior-related measurements were carried out using the Radiant Precision Premier II Ferroelectric Tester (Albuquerque, NM, USA), and the DC leakage measurement were conducted by Keysight B1500A Semiconductor Device Parameter Analyzer (Santa Rosa, CA, USA).

## 3. Results and Discussion

**Figure 2:**
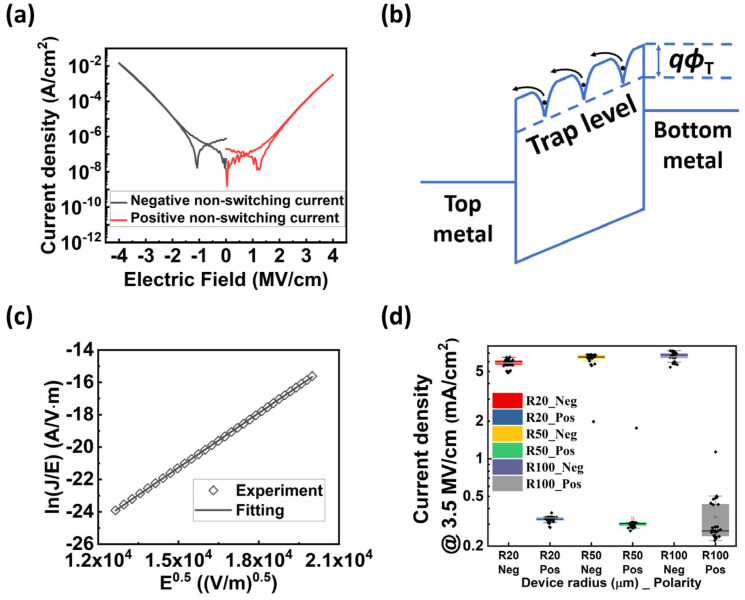
(**a**) Non-switching DC *J-E* curves of the as fabricated device. (**b**) Schematic illustration of P-F emission. (**c**) Fitting of experimental ln(*J*/*E*)-*E*^0.5^ data to the P-F model, showing good linearity. (**d**) Current density vs. device size, showing no obvious size-dependent behavior.

Figure 2a shows the representative non-switching DC *J-E* curves of the as-fabricated capacitor, in which the leakage current is at 10^−3^~10^−2^ A/cm^2^ level at *E* = ±4 MV/cm. The positive polarity refers to the circumstance that applies a positive voltage onto the top electrode, while the negative polarity corresponds to the condition of applying a negative voltage onto the top electrode, respectively. We then investigated the conduction mechanism to understand the origin of the large leakage. The Al_0.7_Sc_0.3_N film was grown by PLD and contained a few point defects, meaning that several trap states exist within the bandgap. In our previous work, we have found that the dominant conduction mechanism of our Al_0.7_Sc_0.3_N films is the P-F emission as illustrated in Figure 2b [34]. The original P-F equation can be expressed by:

(1) $$\;J=q\mu n_{0}Eexp[-\frac{q\phi_{T}-\sqrt{q^{3}E/\pi \varepsilon_{0}\varepsilon_{r}}}{rkT}]$$

where *J* is the current density, *μ* is the carrier mobility, *q* is the elementary charge, *E* is the applied electric field, *qɸ*_T_ (= *Φ*_T_) is the trap energy level, *ε*_0_ is the vacuum permittivity, *ε*_r_ is the optical dielectric constant, *r* is the compensation factor, *k* is the Boltzmann constant, *T* is the absolute temperature [38]. It should be noted that *r* varies from 2 to 1 with the increase of acceptor concentration [38]. For Equation (1), we note that the *ε*_r_ obtained in the equation is the dielectric constant at high frequency, which can be calculated by $\varepsilon_{r}={RI}^{2}$, where RI is the refractive index. The $\varepsilon_{r}$ of the Al_0.7_Sc_0.3_N film used in our calculations is ~4.8 based on literature [39]. We began the P-F analysis by determining the compensation factor based on the *J-E* results at room temperature (Figure 2a). Figure 2b shows the schematic illustration of P-F emission model, which is a trap-assisted conduction mechanism. The ln(*J*/*E*)*-E*^0.5^ curve of the device exhibits a clear linear relationship between ln(*J*/*E*) and *E*^0.5^, following the dependency of the P-F emission, as shown in Figure 2c. The slope obtained from Figure 2c is used for the extraction of the compensation factor, and the *r* is extracted to be ~1.19. This indicates that the as-grown AlScN film is a p-type material [38]. It should be noted that there might be some different conduction mechanisms (i.e., Schottky emission) for AlScN using different growing methods or with different Sc doping concentration [35]. However, the extracted optical dielectric constant of our film using the Schottky emission model is ~1.39, which is unreasonably smaller than the experimental value (~4.8) [39], indicating the Schottky emission is not the major mechanism for our devices (see Supplementary Materials for more details). Additionally, Figure 2d plots the statistical results of the leakage current at *E* = ±3.5 MV/cm of the devices with different pad sizes. There is no obvious size-dependent behavior, indicating that the leakage is not contributed to by the conducting filaments.

To further identify the type of traps leading to the leakage current, the density functional theory (DFT) calculations were performed. The DFT calculations were carried out utilizing the pw.x package in Quantum Espresso (QE) and the PBE exchange–correlation functionals for the Al, N, and Sc atoms were employed [40,41]. The crystal model ${Al}_{7}{Sc}_{0.3}N$ was constructed using the Special Quasi-Random Structure method [42,43,44]. For the relaxation process, a Monkhorst–Pack mesh with the dimensions of k-points as 6 × 6 × 4 was set. The PAW (projector augmented wave) was utilized as pseudopotentials and structural relaxation was carried out using the Broyden–Fletcher–Goldfarb–Shanno algorithm with Gaussian smearing of 0.01 Ry. The total energy convergence threshold was established at 10^−6^ Ry and the cut-off energy of 40 Ry was selected for the plane-wave basis set. Subsequently, a more refined k-point grid of 8 × 8 × 5 was utilized for total energy calculations.

A defect (*x*) in a charge state *q* state’s formation energy $E_{form}\left(X,q\right)$ is defined as follows:

(2) $$E_{form}\left(X,q\right)=E_{defect}\left(X,q\right)-E_{perfect}\left({Al}_{0.7}{Sc}_{0.3}N\right)-\sum_{i}n_{i}u_{i}+q\left(E_{F}+E_{VBM}\right),$$

where the $E_{perfect}({Al}_{0.7}{Sc}_{0.3}N)$ is the total energy of the pristine composition Al_0.7_Sc_0.3_N model. $E_{defect}(X,q)$ is total energy of the model removed from Al, Sc, or N atom in a *q* charge state [45]. In addition, the term $n_{i}u_{i}$ represents the energy of the corresponding crystalline reservoir of Al, Sc, or N, as well as the number of vacancies formed when an atom is removed [45]. *E*_F_ represents the Fermi energy level relative to the energy position (*E*_VBM_) of the VBM in the perfect supercell (${Al}_{0.7}{Sc}_{0.3}N$). The summation term is the contribution of the chemical potential of each atomic species on the formation energy of the defect.

**Figure 3:**
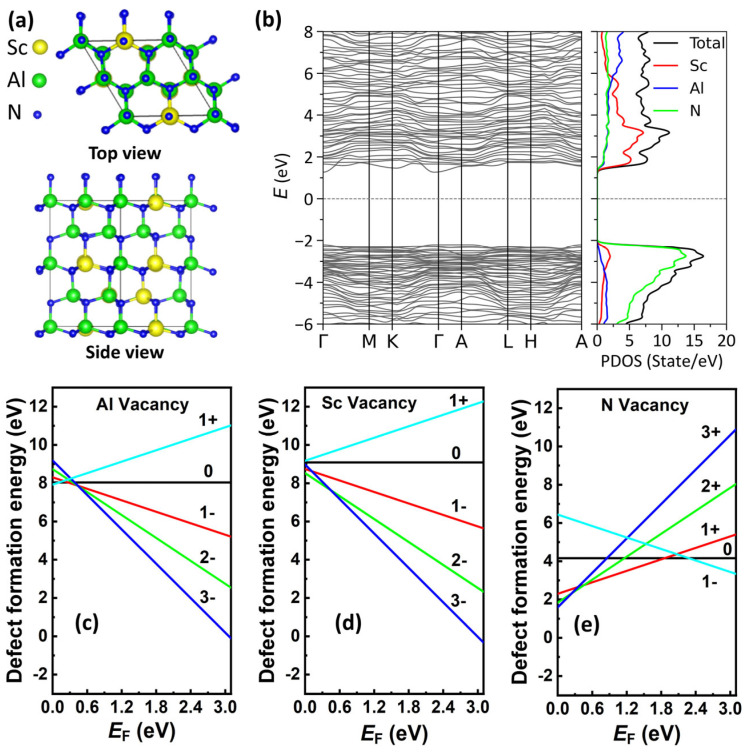
(**a**) Snapshots of the top and side views of the wurtzite Al_0.7_Sc_0.3_N model. (**b**) The band structure of the perfect cell of Al_0.7_Sc_0.3_N model. The Fermi level is set to zero and represented by the horizontal dashed line. (**c**–**e**) Defect formation energies of Al_0.7_Sc_0.3_N models with different types of vacancy $V_{N}$, $V_{Sc}$, and $V_{Al}$ in the relaxed configurations as a function of Fermi level. *E*_F_ = 0 corresponds to VBM.

Figure 3a shows the relaxed geometry of 32-atom wurtzite Al_0.7_Sc_0.3_N supercells. Figure 3b reveals the band structure and density of states (DOS) of the perfect cell wurtzite Al_0.7_Sc_0.3_N model with fully geometry relaxation. The calculated bandgap of the pristine Al_0.7_Sc_0.3_N model is ~3.28 eV as shown in Figure 3b, which agrees well with experimental values [46]. Figure 3c–e discloses defect formation energies as a function of Fermi level (*E*_F_) of the wurtzite Al_0.7_Sc_0.3_N model for different types of vacancies, including aluminum vacancy (*V*_Al_), scandium vacancy (*V_Sc_*), and nitrogen vacancy (*V*_N_), with different charge states. The overall smallest formation energies resulting from every charge state (from 3- to 1+ charge state for *V*_Al_ and *V_Sc_*, and from 1- to 3+ charge state for *V*_N_) are exhibited. In the low *E*_F_ region (i.e., p-type case), the Al_0.7_Sc_0.3_N model with *V*_N_ shows smaller formation energies than other types of vacancies. On the other hand, the systems with a triplet negatively charged aluminum vacancy ($V_{Al}^{3-}$) or scandium vacancy ($V_{Sc}^{3-}$) exhibit low formation energies in an n-type case, which agree well with the previous native AlN defects. DFT results indicate that the *V*_N_ in AlN has the lowest formation energy in p-type material and the *V*_Al_ has the lowest formation energy in n-type material [47,48,49,50]. The experimental results demonstrate that the as-grown AlScN is a p-type material, suggesting that the leakage current is caused by the generation of *V*_N_ during thin film deposition, rather than *V*_Al_ or *V_Sc_*. Especially, *V*_N_ with positive charge states ($V_{N}^{1+},V_{N}^{2+},V_{N}^{3+}$) shows the smallest formation energy at the low *E*_F_ region, indicating the highest possibility to be formed.

**Figure 4:**
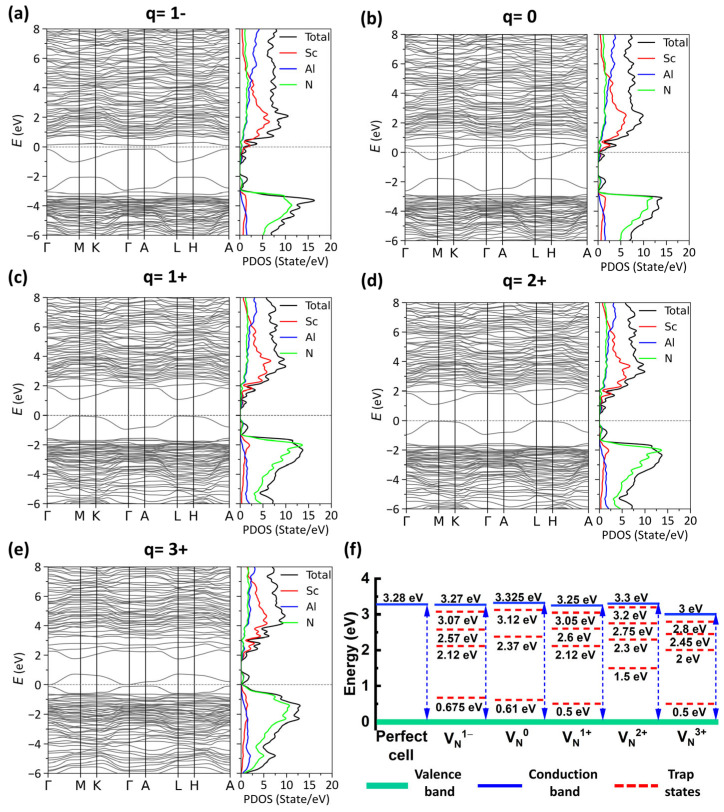
(**a**–**e**) Band gap structures of the Al_0.7_Sc_0.3_N with one nitrogen vacancy at different charge states. (**f**) Extracted band gap and defect states of the perfect Al_0.7_Sc_0.3_N and Al_0.7_Sc_0.3_N with one nitrogen vacancy at different charge states.

Subsequently, the electronic band structure for the Al_0.7_Sc_0.3_N model with one *V*_N_ at different charge states was calculated, as shown in Figure 4a–e. It is observed that the Fermi level is closer to the valence band (p-type) when there exists $V_{N}^{1+}/V_{N}^{2+}/V_{N}^{3+}$ compared with $V_{N}^{1+}/V_{N}^{0}\;$. This result confirms that the *V*_N_ is dominated by the vacancies with positive charge states in the p-type AlScN film. The band gaps and the defect states induced by different *V*_N_ vacancies are summarized as shown in Figure 4f. It is observed that the nitrogen vacancies will induce multiple defect states near both the conduction band and valence band, which might facilitate the P-F emission. Considering the experimental results and theoretical calculation, the large leakage in the as-grown p-type Al_0.7_Sc_0.3_N film might originate from the *V*_N_ with positive charge states and follows the conduction mechanism of P-F emission.

**Figure 5:**
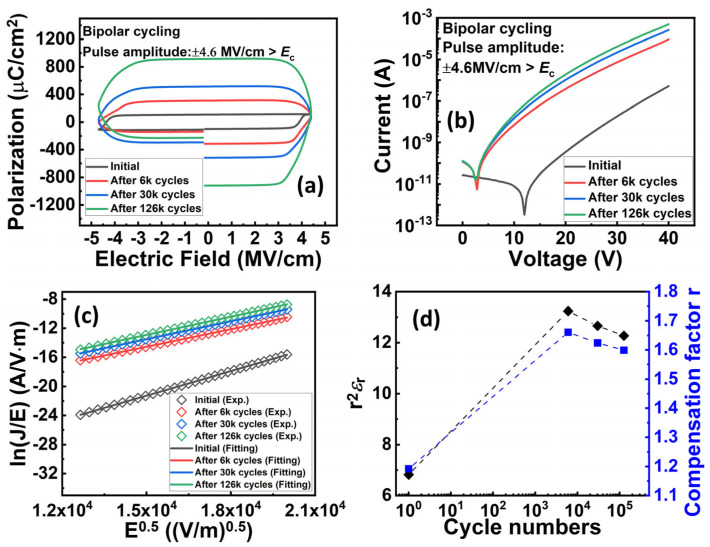
(**a**,**b**) *P-E* loops and DC *I-V* curves of the devices undergoing bipolar cycling tests at room temperature, showing a significant leakage current increment after the cycling. (**c**) Fitting of experimental ln*(J/E)-E*^0.5^ data to the P-F model, showing good linearity. (**d**) Extracted *r*^2^*ε*_r_ and compensation factor vs. bipolar cycling numbers.

**Figure 6:**
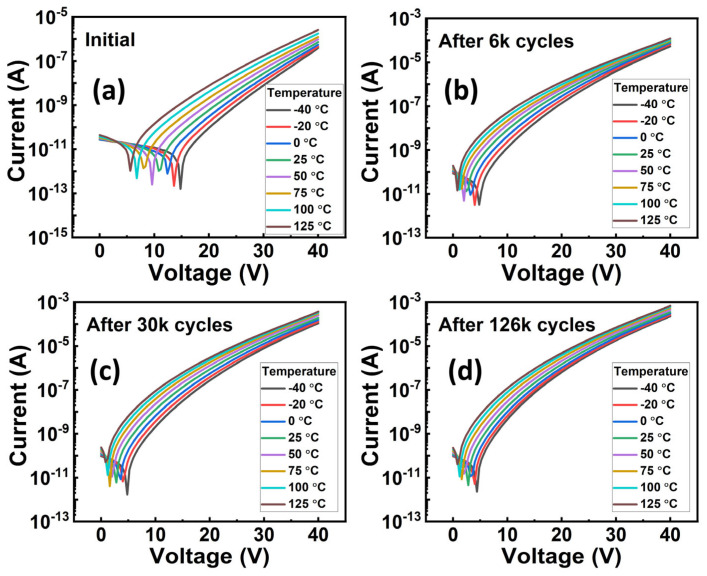
Temperature-dependent *I-V* curves of the device undergoing bipolar cycling with a pulse amplitude of ±4.6 MV/cm. Initial *I-V* curves (**a**), *I-V* curves after 6 k (**b**), 30 k (**c**), and 126 k cycles (**d**), respectively.

**Figure 7:**
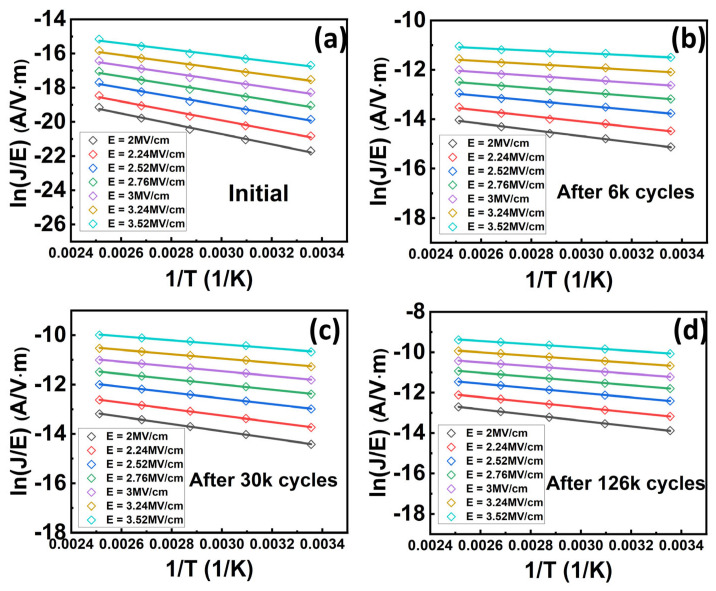
Fitting of experimental ln(*J*/*E*)-1*/T* data to the P-F model of initial device (**a**), after 6 k (**b**), 30 k (**c**), and 126 k cycles (**d**), showing good linearity in all conditions.

**Figure 8:**
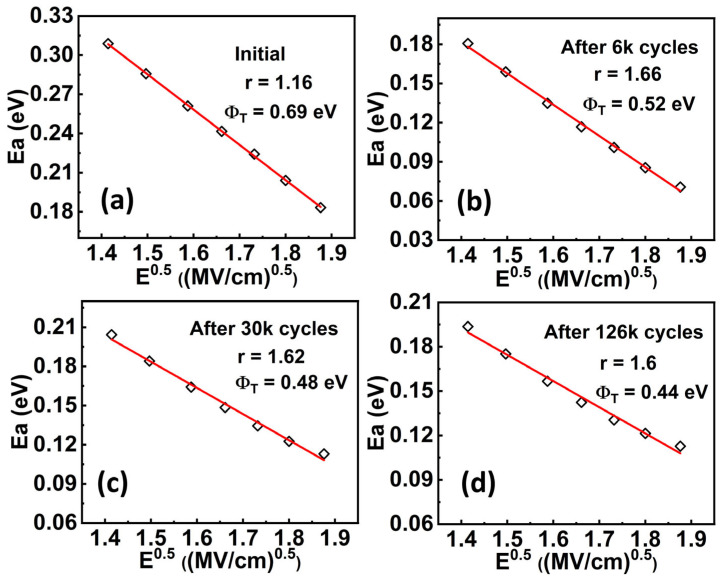
Fitting of experimental *E*_a_-*E*^0.5^ data to the P-F model of initial device (**a**), after 6 k (**b**), 30 k (**c**), and 126 k cycles (**d**), showing good linearity in all conditions.

**Figure 9:**
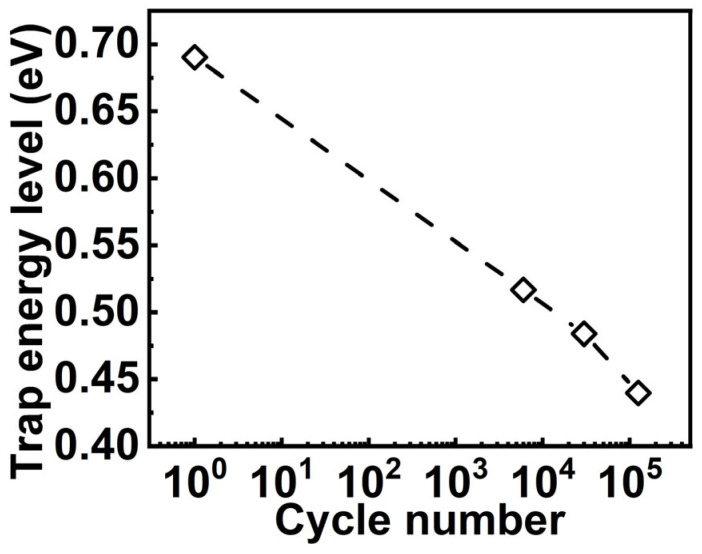
Extracted trap energy level vs. bipolar cycling numbers.

Cycling behavior is another essential property of using this material for practical memory applications. We then characterized the cycling behavior of the Al_0.7_Sc_0.3_N-based capacitors. Specifically, bipolar cycling tests by applying a series of consecutive bipolar waves onto the capacitor were performed and *P-E* loops were measured subsequently. The bipolar cycling waves used in this work are square pulses with a frequency of 2 kHz, and a pulse width of 12 µs (See Supplementary Materials for more details), respectively. It is observed that bipolar cycling with a pulse amplitude (±4.6 MV/cm) larger than the *E*_c_ results in a distorted *P-E* loop, indicating a significant increase of the leakage current which will cause a read disturbed issue for the FeRAM devices (Figure 5a). However, there shows no obvious degradation when either unipolar pulses with a pulse amplitude larger than *E*_c_ or bipolar pulses with a pulse amplitude smaller than *E*_c_ were applied (see Supplementary Materials for more details). This indicates that the increment in leakage requires the polarization state to be switched. In our previous work, we found that the bipolar cycling would lead to grain fragmentation, but we did not deeply link it to the impact of traps [37]. In this work, after confirming the trap-assisted conduction mechanism and specific type of traps in Al_0.7_Sc_0.3_N by the above-mentioned experiment and theoretical calculation, we then analyzed the impact of traps on the cycling behavior of Al_0.7_Sc_0.3_N. We conducted DC leakage current measurements after cycling to verify our observations in the *P-E* measurements and analyzed the impact of traps after cycling. Our DC *I-V* measurements indicate concordance with our *P-E* measurements (Figure 5b), in which the device undergoing bipolar cycling with a pulse amplitude (±4.6 MV/cm) larger than *E*_c_ can trigger a substantial leakage increment. In the meanwhile, all the ln(*J*/*E*)–*E*^0.5^ curves of the device undergoing bipolar cycling exhibit a clear linear relationship between ln(*J*/*E*) and *E*^0.5^ (Figure 5c), agreeing with the dependency of the P-F emission. Then, the slopes obtained from Figure 5c are used for the extraction of the compensation factor (Figure 5d), which will be utilized for extracting the trap energy level subsequently. The *r* is extracted to be ~1.19, 1.66, 1.62, and 1.6 for the initial state of the device, and when it undergoes 6000, 30,000, and 126,000 cycles of bipolar cycling, respectively. The extracted *r* increases substantially after bipolar cycling, indicating that the Al_0.7_Sc_0.3_N is a less p-type material and its Fermi level shifts to the conduction band. Furthermore, the temperature-dependent DC *I-V* measurements (from −40 °C to 125 °C, with a step of 25 °C) after undergoing different cycles of bipolar cycling were taken to extract the trap energy level (Figure 6a–d). The ln(*J*-*E*)-1*/T* and *E*_a_*-E*^0.5^ curves show clear linearity under all conditions (Figure 7 and Figure 8), where $E_{a}=q\phi_{T}-\sqrt{q^{3}E/\pi \varepsilon_{0}\varepsilon_{r}}$. By taking the compensation factor into consideration, the trap energy level extracted from the temperature-dependent DC *I-V* results would decrease from 0.69 eV to 0.44 eV monotonously after 10^5^ switching cycles (Figure 9), which should be the cause of leakage increment.

## 4. Conclusions

In summary, the reliability properties of the Al_0.7_Sc_0.3_N, including the origin of the leakage and cycling behavior of Al_0.7_Sc_0.3_N-based FeRAM device, are experimentally and theoretically investigated. The findings indicate that the leakage can be traced back to nitrogen vacancies with positive charge states, and it follows the conduction mechanism of P-F emission. Additionally, bipolar cycling tests reveal a significant increase in leakage when the capacitor undergoes cycles with a pulse amplitude larger than the *E*_c_, which is caused by the reduced trap energy level after the bipolar cycling.

## Data Availability

Data are contained within the article.

## Supplementary Materials

The supporting information can be downloaded at: https://www.mdpi.com/article/10.3390/ma17020397/s1, Figure S1: Schematic of the pulse setup for cycling tests; Figure S2: Schottky emission fitting; Figure S3: *P*-*E* loops of the devices undergoing bipolar cycling with a pulse amplitude smaller than *E*_c_ and unipolar cycling with a pulse amplitude larger than *E*_c_.
