# Supplementary material for: Leakage Mechanism and Cycling Behavior of Ferroelectric Al0.7Sc0.3N
Source: Materials (Basel). 2024 Jan 12;17(2):397. doi: 10.3390/ma17020397 (PMC10817578; doi:10.3390/ma17020397)
Supplement: Supplementary file 1 [file materials-17-00397-s001.zip › materials-2764374-supplementary.pdf]

Supporting Information

Leakage Mechanism and Cycling Behavior of Ferroelectric $Al_{0.7}Sc_{0.3}N$

Li Chen [1,†], Qiang Wang [2,†], Chen Liu [1,*], Minghua Li [1], Wendong Song [1], Weijie Wang [1], Desmond K. Loke [2,*] and Yao Zhu [1,*]

[1] Institute of Microelectronics, Agency for Science, Technology and Research (A*STAR), Singapore 138634, Singapore; chen_li@ime.a-star.edu.sg (L.C.); li_minghua@ime.a-star.edu.sg (M.L.); song_wen_dong@ime.a-star.edu.sg (W.S.); wang_weijie@ime.a-star.edu.sg (W.W.)

[2] Department of Science, Mathematics and Technology, Singapore University of Technology and Design, Singapore 487372, Singapore; wang_qiang@ihpc.a-star.edu.sg

* Correspondence: liu_chen@ime.a-star.edu.sg (C.L.); desmond_loke@sutd.edu.sg (D.K.L.); zhuya@ime.a-star.edu.sg (Y.Z.)

† These authors contributed equally to this work.

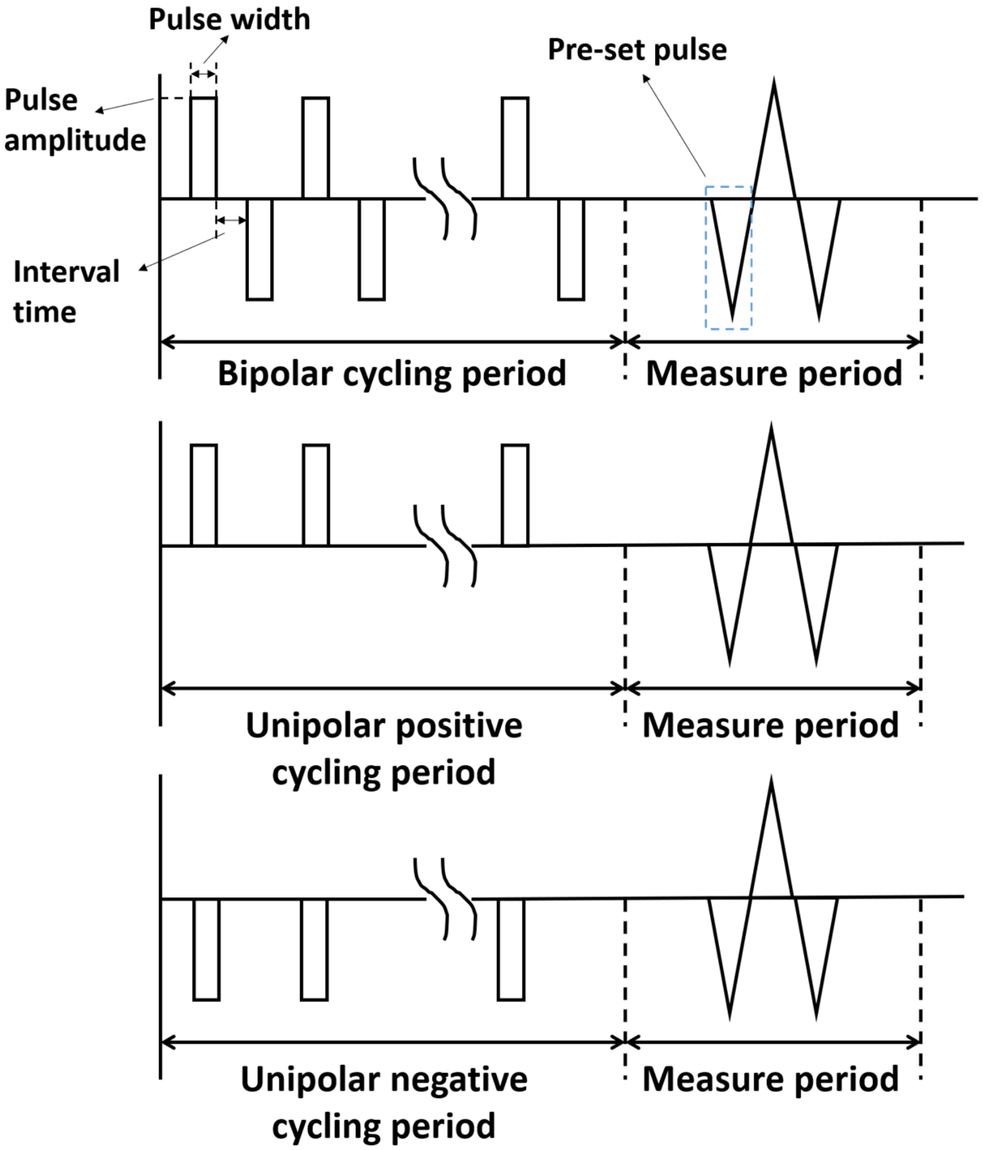

Figure S1. Schematic of the pulse setup for cycling tests.

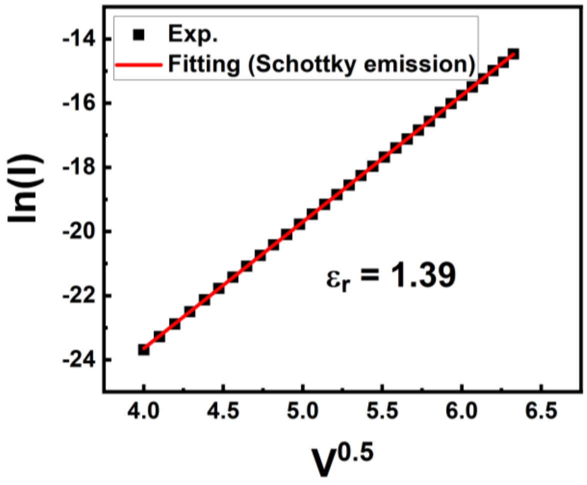

Figure S2. Fitting of experimental ln(I)-$V^{0.5}$ data to the Schottky emission model. The extracted optical dielectric constant is 1.39.

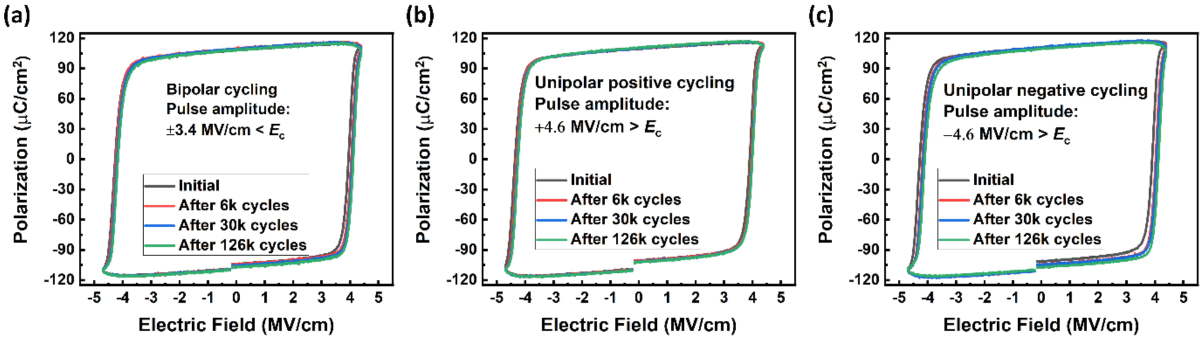

Figure S3. *P*-*E* loops of the devices undergoing bipolar cycling with a pulse amplitude smaller than E_c (a), and unipolar cycling with a pulse amplitude larger than E_c (b)-(c), respectively. There are no obvious *P*-*E* degradation for all these three cases.
